# Whole genome analysis of *Shigella* sp. JZ001: a novel strain isolated from diarrheic suckling mice

**DOI:** 10.3389/fvets.2025.1686554

**Published:** 2025-11-21

**Authors:** Xiang yu Li, Piao Piao Yang, Yunzhu Tang, XiaoFeng Li, Xuejiao Jia, Mengqi Liu, Yonggang Li, Wei Zhao

**Affiliations:** 1 College of Basic Medical Sciences, Jinzhou Medical University, Jinzhou, China; 2 Liaoning Province Key Laboratory of Human Phenome Research, Jinzhou, China

**Keywords:** *Shigella* spp., diarrheic suckling mice, virulence, whole genome sequencing, comparative genomic analysis

## Abstract

**Background:**

*Shigella* spp. are Gram-negative enteropathogens responsible for bacillary dysentery (shigellosis) in humans and animals. While the primary reservoirs of *Shigella* are historically recognized as humans and non-human primates, emerging evidence indicates an expanding host range encompassing diverse animal species. This study systematically characterizes a *Shigella* sp. strain isolated from diarrheic suckling mice.

**Methods:**

Diarrheic suckling mice intestinal contents were collected and analyzed using 16S rRNA sequencing technology. *Shigella* sp. JZ001 strain was isolated, purified, and identified. Bacterial morphology was examined by transmission electron microscopy. Carbon source utilization profiles were determined using BIOLOG GENIII MicroPlates™. Short-chain fatty acid (SCFA) composition was quantified by gas chromatography–mass spectrometry (GC–MS) following methyl-tert-butyl ether extraction. Polar lipid analysis was performed through two-dimensional thin-layer chromatography with detection using 10% ethanolic molybdatophosphoric acid. Whole genome sequencing was performed using a hybrid approach combining Illumina NovaSeq and Nanopore MinION platforms. Phylogenetic reconstruction was performed using maximum-likelihood algorithms implemented in MEGA.

**Results:**

Rotavirus-SA11 infected suckling mice exhibited watery feces 4 days post-infection. Intestinal contents were analyzed using 16S rRNA sequencing technology, which revealed a significant increase in the genus *Shigella* in the RV-infected group. A *Shigella* strain was isolated and purified from the intestinal contents and designated as JZ001. Polyphasic characterization confirmed that strain JZ001 belongs to the Enterobacteriaceae family and is phylogenetically closest to *Shigella* spp. The genome consists of a single circular chromosome (5,329,126 bp; G + C content 50.65%) and one plasmid (1,09829 bp, G + C content 40.14%). Comparative genomic analysis identified 24 Salmonella pathogenicity island (SPI)-like regions. Substrate assimilation tests demonstrated metabolic versatility, including utilization of D-Maltose, D-Trehalose, *α*-D-Glucose, D-Mannose, D-Fructose, D-Galactose and Propionic Acid. Cellular fatty acid methyl ester (FAME) analysis identified predominant components as: Dodecanoic acid (12:0; 11.08%), Tetradecanoic acid (14:0; 12.00%), Hexadecanoic acid (16:0; 21.55%), cycloheptadecanoic acid (17:0 cyclo; 13.99%), Dodecanal (12:0 aldehyde; 22.32%). The polar lipid profile consisted of diphosphatidylglycerol, phosphatidylglycerol, and phosphatidylethanolamine, with notable absence of aminolipids and aminophospholipids.

**Conclusion:**

First study of murine *Shigella* JZ001 offers insights into genomic plasticity and host adaptation mechanisms. The genomic dataset lays foundation for host-pathogen studies and facilitates developing murine models for therapy evaluation.

## Introduction

Shigellosis is an acute inflammatory bowel disease characterized by bloody mucoid diarrhea, pyrexia, and abdominal cramping, typically presenting as a self-limiting gastrointestinal infection. The etiological agent, *Shigella* spp., are Gram-negative, non-sporulating bacilli within the family *Enterobacteriaceae* (1). Human-pathogenic *Shigella* strains are taxonomically classified into four species: *S. dysenteriae* (serogroup A), *S. flexneri* (serogroup B), *S. boydii* (serogroup C), and *S. sonnei* (serogroup D) (2). Clinical manifestations in humans include colonic mucosal ulceration, acute-phase responses (leukocytosis, elevated C-reactive protein), and electrolyte imbalance secondary to secretory diarrhea (3). In contrast, the pathophysiological presentation of shigellosis in non-human hosts remains poorly characterized (4). The natural hosts of *Shigella* are typically humans and other primates, but it has been shown that the host range of *Shigella* has expanded to many animals (5–9). Animal *Shigella* infection is becoming a major threat to animal health and a potential zoonotic risk, posing dangers of cross-species transmission to humans. However, limited information is available regarding the genetic background of *Shigella* strains isolated from animals. Previous reports indicate that mice are naturally resistant to oral challenge with *Shigella* (10). The lack of a suitable animal model has significantly hindered our understanding of *Shigella* pathogenesis. To date, no murine-derived *Shigella* strains have been reported, particularly with respect to their isolation and whole-genome architecture.

In this study, we isolated a novel *Shigella* strain (designated JZ001) from diarrheic laboratory-bred suckling mice and conducted systematic pheno-genomic characterization. Through comparative whole-genome sequencing and functional annotation, we revealed close phylogenetic relationships with human clinical isolates. These findings offer critical insights into the evolutionary trajectory of *Shigella* across host species boundaries and provide foundational data for developing murine infection models to study zoonotic transmission dynamics.

## Methods

### Collection and treatment of sample and 16S rRNA sequencing

Three-day-old suckling mice were divided into two groups: Rotavirus SA11 infected group and uninfected controls group. The RV-infected group was orally administered 50 μL of RV strain SA-11 at a concentration of 10^6^ PFU/mL, while the uninfected control group received 50 μL of phosphate-buffered saline (PBS) (11). All mice were euthanized 4 days post-RV infection, at the time point when the most severe diarrheal symptoms were observed. The procedures for the care and use of animals were approved by the Committee on Laboratory Animal Ethics of Jinzhou Medical University, and all applicable institutional and governmental regulations concerning the ethical use of animals were followed (Approval ID: 2019014).

16S rRNA gene sequencing and analysis were performed as described in our previous study (11). Briefly, genomic DNA was extracted from mouse fecal samples. The V3–V4 region of the bacterial 16S ribosomal RNA gene was amplified using primers 342F and 806R, and sequenced on the Illumina MiSeq platform (Illumina, Inc., San Diego, CA, USA). The sequencing reads were assembled and used for subsequent 16S analysis. Based on phylotypic annotation, the relative abundances of the top 10 genera were calculated, and star-plot histograms were generated for each sample at the genus level. Phylotypes with relatively high abundances and their proportions could be examined across different taxonomic levels (12, 13).

Fecal samples were also diluted with sterile PBS and streaked onto Salmonella–Shigella (SS) agar plates. Twenty bacterial colonies were randomly selected for PCR detection and sequencing analysis. Colonies testing positive for *Shigella* spp. were subsequently re-cultured on SS agar for further characterization.

### Culture media, bacterial isolation and cultivation

The faecal specimens were streaked onto selective agars for the isolation of *Shigella* spp. as described previously (14). The *Shigella* isolates were identified biochemically using triple-sugar iron, lysine iron, motility-indole-ornithine, and Simmons citrate agars. Colonies appearing after incubation for 2, 5 and 10 days were picked and re-streaked on agar plates of same media. Bacterial purity was evaluated by by assessing colony morphology, 16S rRNA gene and genome sequencing.

### Cell morphology observation and chemotaxonomic determinations

Single colonies of *Shigella* were inoculated and cultured overnight at 37 °C. 1 mL of the resulting culture was harvested, and bacterial cells were collected by centrifugation at 8,000 × g for 10 min at 4 °C. Subsequently, 10 μL of the resuspended bacterial suspension was dropped onto a copper grid. 10 μL of 1.5% phosphotungstic acid solution (pH 6.8–7.0) was added to the grid for staining, with the staining process lasting 90 s. Following staining, the grid was air-dried at room temperature for 15–20 min and finally observed under a transmission electron microscope (HT7800, HITACHI).

Carbon sources utilization was determined using the 96-well BIOLOG GENIII MicroPlate (OmniLog Data Collection Software, Identification System version 2.3, BIOLOG), which contained 95 different carbon substrates. Bacteria strains were cultured in TSA medium for 24 h at 30 °C, after which the cells were harvested. Cellular fatty acids were then extracted and methylated according to the standard midi protocol by Agilent 6,890 N (Fatty Acid Methyl Esters, Microbial ID, Sherlock Version 6.0B). Identification was performed by SHERLOCK® Microbial Identification System (Microbial ID, American). Short-chain fatty acids (SCFAs) were measured using GC–MS. For extraction, 1 mL of culture was mixed with 1 mL of ethyl acetate, and the upper layer was collected for GC–MS analysis. The separation of polar lipids was performed using two-dimensional thin-layer chromatography (TLC) on silica gel coated plates, 10×10 cm; Merck. The detection of total lipds was accomplished by employing a solution of 10% ethanolic molybdate and phosphoric acid (Sigma). Aminolipids were detected using a 0.4% solution of ninhydrin (Sigma) in butanol. The presence of phospholipids was detected using a Zinzadze reagent (a molybdenum blue spray reagent with a concentration of 1.3%, obtained from Sigma). The identification of glycolipids was accomplished through the utilization of a 0.5% *α*-naphthol sulphuric acid reagent.

### Phylogenetic tree construction

Phylogenetic analysis was performed using the ClustalW alignment tool and the Molecular Evolutionary Genetic Analysis (MEGA X) software^1^ (15).

### Whole genome sequencing and analysis

Genomic DNA was extracted using the Bacteria DNA Kit (Tiangen Biotech Co., Ltd., Beijing, China). DNA integrity and purity were assessed using 1% agarose gel electrophoresis. The Qubit 4.0 Fluorometer and NanoDrop One spectrophotometer (both from Thermo Fisher Scientific, Waltham, USA) were used to determine DNA concentration and purity.

DNA template libraries were constructed using the BluePippin system, with concentration measured by Qubit 4.0 and average fragment size evaluated using the Agilent 4,200 system (Agilent Technologies, Santa Clara, CA) (16). Genome component prediction included coding genes, repetitive sequences, non-coding RNAs, and prophages. Gene prediction was performed using Glimmer3 (17). Transfer RNAs (tRNAs) were predicted using tRNAscan-SE (version 1.4; http://lowelab.ucsc.edu/tRNAscan-SE/), while ribosomal RNAs (rRNAs) were identified using rRNAmmer (version 1.2; http://www.cbs.dtu.dk/services/RNAmmer/). Small RNAs (sRNAs) were annotated by comparison with the Rfam database and filtered using the cmsearch program (18–20).

Prophage prediction was carried out using PHAST_finder (version 2.1) (21), and genomic islands were identified with IslandPath-DIMOB (22). CRISPR sequences were detected using CRISPRdigger (23).

Functional annotation of predicted genes was performed using whole-genome BLAST searches against the NR (Non-Redundant Protein), COG (Clusters of Orthologous Groups), Swiss-Prot, KEGG (Kyoto Encyclopedia of Genes and Genomes), and GO (Gene Ontology) databases. Prediction of secreted proteins was conducted using SignalP (24), LipoP (25), TMHMM (26), and PSORTb (27). Pathogen-host interaction genes were annotated using the PHI database (28), and virulence factors were identified using the VFDB (29).

Based on genome assembly, coding gene prediction, non-coding RNA analysis, and functional annotations, the complete genome map was visualized using Circos software (30).

The complete genome sequence of strain JZ001 has been submitted to the National Center for Biotechnology Information (NCBI) under BioProject number PRJNA1238207.

### Comparative genomic analysis

Ten publicly available genomes of *Shigella* sp.—including GCF_002290485.1, GCF_022354085.1, GCF_013374815.1, GCF_002950275.1, GCF_002950135.1, GCF_002950295.1, GCF_002950215.1, GCF_000754175.1, GCF_002950155.1, and GCF_002950055.1—were utilized for comparative genomic analysis with strain JZ001. Orthologous Average Nucleotide Identity Software Tool (OAT) (31) was employed to ascertain the average nucleotide identity (ANI) of 16S rRNA, with the objective of identifying bacterial genomic relatives. ANI > 95% indicates that the two genomes belong to the same species. MUMmer software (Version 3.23) was then employed to compare the *Shigella Sp* JZ001 genome with the reference genome (32), and the collinearity relationship between the genomes was determined. Subsequently, gene family analysis was conducted using the OrthoFinder program with protein sequences from 11 genomes as input files (33).

### Culture preservation

Bacterial strains were cultured in liquid medium for 2 days. The cultures were stored in the laboratory by the addition of an equal volume of 65% (v/v) glycerol (1 mL), and then stored at −80 °C for long-term preservation. All type strains assigned by this study were deposited at the Agricultural Culture Collection of China (ACCC) under the number ACCC 64336.

## Results

### Isolation *Shigella* in mice

**Figure 1:**
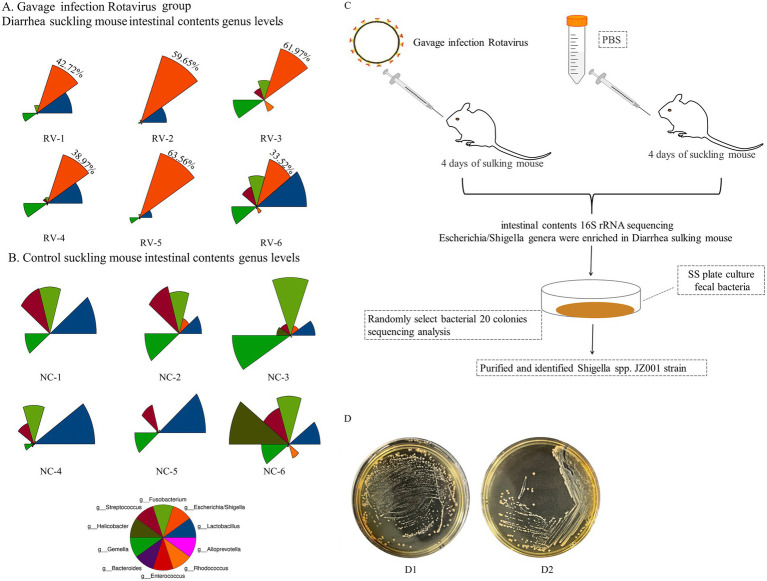
**(A,B)** Show the results of intestinal microbiota analysis on fecal samples from rotavirus-infected diarrheic suckling mice (RV group) and age-matched control mice (NC group). **(A)** The abundance of bacterial phylotypes in the gut microbiota of fecal samples from diarrheic mice (RV group), The red color sector represents *Shigella* spp., and its relative percentage values are displayed in the red sector area; **(B)** control mice (NC group). The relative abundances of the top 10 bacterial phylotypes at the genus level via star plots (Supplementary Table S1). Star plots visualize the relative abundance of individual phylotypes within each sample: each sector, distinguished by a unique color, corresponds to one genus-level phylotype, and the radius of each sector is proportional to the relative abundance of the corresponding phylotype—with a longer radius indicating a higher relative abundance. **(C)** Schematic diagram of isolation method for *Shigella* spp. in mice. **(D)** D1 Mice fecal culture on SS agar plate; D2 *Shigella* spp. Culture in SS plate.

In this study, intestinal microbiota analysis was conducted on previously collected samples from rotavirus-infected diarrheic suckling mice (11). As shown in Figures 1A,B, the fecal microbiota of rotavirus-infected mice was dominated by *Lactobacillus* spp. and *Shigella* spp. Notably, the relative abundance of *Shigella* spp. in the rotavirus-infected group was markedly higher than in the uninfected control group (Figure 1B). The detailed relative abundance values of all microbial taxa identified in the community composition analysis are provided in Supplementary Table S1.

Based on these findings, diarrheal fecal samples were first cultured on SS agar. Twenty randomly selected colonies were analyzed, and two were identified as *Shigella* spp. (Figure 1C). Subsequently, targeted isolation was conducted using *Shigella*-selective agar, and colonies confirmed as *Shigella* sp. JZ001 by PCR and sequence analysiswere selected for pure culture (Figure 1D).

### Bacterial growth and cell morphology

**Figure 2:**
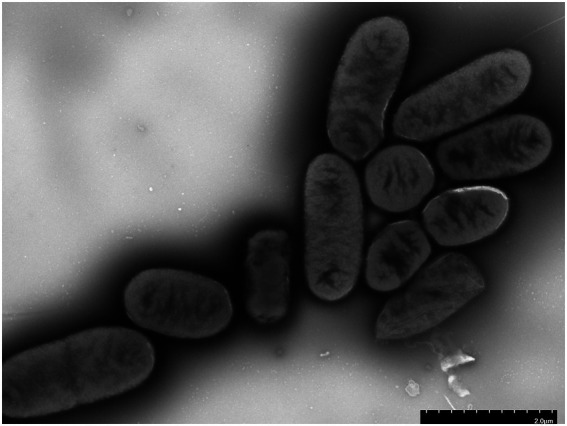
Representative transmission electron microphotographs (TEM) of JZ001 strain Overnight culture of each strains were collected and stained by with 1.5% phosphotungstic acid for 90 s and examined under TEM. Magnification = 15,000.

After 24 h of cultivation on standard agar medium, *Shigella* strain JZ001 formed semi-transparent, smooth-surfaced, circular, gray-white colonies. Examination of cell morphology (Figure 2) revealed that JZ001 cells were rod-shaped and lacked flagella.

### Physiological and biochemical characterization

The assimilation of 95 carbon sources by strain JZ001 was assessed, with the results summarized in Table 1. The strain metabolized 64 of the 95 carbon sources tested, showing a broad metabolic versatility in carbon source utilization. Monosaccharides and disaccharides were particularly favored. Notably, JZ001 was able to assimilate D-maltose, D-trehalose, *α*-D-flucose, D-mannose, D-fructose, D-galactose, and propionic acid, demonstrating a versatile metabolic profile. The JZ001 strain was capable of growth under both acidic (pH 5) and weakly acidic (pH 6) conditions. It also grew stably in the presence of 1, 4, and 8% NaCl and exhibited tolerance or reduced sensitivity to most antibiotics and metabolic inhibitors. Notably, the strain demonstrated resistance to rifampicin and vancomycin, among other antibiotics.

**Table 1:** Utilization of the carbon sources on BIOLOG GENIII MicroPlate.

Characteristic	Utilize	Characteristic	Utilize +/-
Negative Control	−	Rifamycin SV	+
Dextrin	+	Minocycline	W
D-Maltose	+	Gelatin	−
D-Trehalose	+	Glycyl-L-Proline	+
D-Cellobiose	−	L-Alanine	+
Gentiobiose	−	L-Arginine	−
Sucrose	+	L-Aspartic Acid	+
D-Turanose	−	L-Glutamic Acid	+
Stachyose	−	L-Histidine	−
Positive Control	+	L-Pyroglutamic Acid	−
pH 6	+	L-Serine	+
pH 5	+	Lincomycin	+
D-Raffinose	+	Guanidine HCl	+
α-D-Lactose	−	Niaproof 4	+
D-Melibiose	+	Pectin	−
β-Methyl-DGlucoside	+	D-Galacturonic Acid	+
D-Salicin	−	L-Galactonic Acid Lactone	+
N-Acetyl-DGlucosamine	W	D-Gluconic Acid	+
N-Acetyl-β-DMannosamine	+	D-Glucuronic Acid	+
N-Acetyl-DGalactosamine	+	Glucuronamide	+
N-Acetyl Neuraminic Acid	+	Mucic Acid	−
1% NaCl	+	Quinic Acid	−
4% NaCl	+	D-Saccharic Acid	+
8% NaCl	+	Vancomycin	+
α-D-Glucose	+	Tetrazolium Violet	+
D-Mannose	+	Tetrazolium Blue	+
D-Fructose	+	p-Hydroxy-Phenylacetic Acid	+
D-Galactose	+	Methyl Pyruvate	−
3-Methyl Glucose	−	D-Lactic Acid Methyl Ester	+
D-Fucose	−	L-Lactic Acid	+
L-Fucose	+	Citric Acid	−
L-Rhamnose	+	α-Keto-Glutaric Acid	−
Inosine	+	D-Malic Acid	+
1% Sodium Lactate	+	L-Malic Acid	+
Fusidic Acid	+	Bromo-Succinic Acid	+
D-Serine	+	Nalidixic Acid	+
D-Sorbitol	+	Lithium Chloride	+
D-Mannitol	W	Potassium Tellurite	+
D-Arabitol	−	Tween 40	−
myo-Inositol	−	γ-Amino-Butryric Acid	−
Glycerol	+	α-Hydroxy-Butyric Acid	+
D-Glucose-6-PO4	+	β-Hydroxy-D, L-butyric Acid	−
D-Fructose-6-PO4		α-Keto-Butyric Acid	W
D-Aspartic Acid	+	Acetoacetic Acid	−
D-Serine	−	Propionic Acid	+
Troleandomycin	+		

+, positive; −, Negative; w, Weakly positive.

### Cellular fatty acid and polar lipid profiling

**Figure 3:**
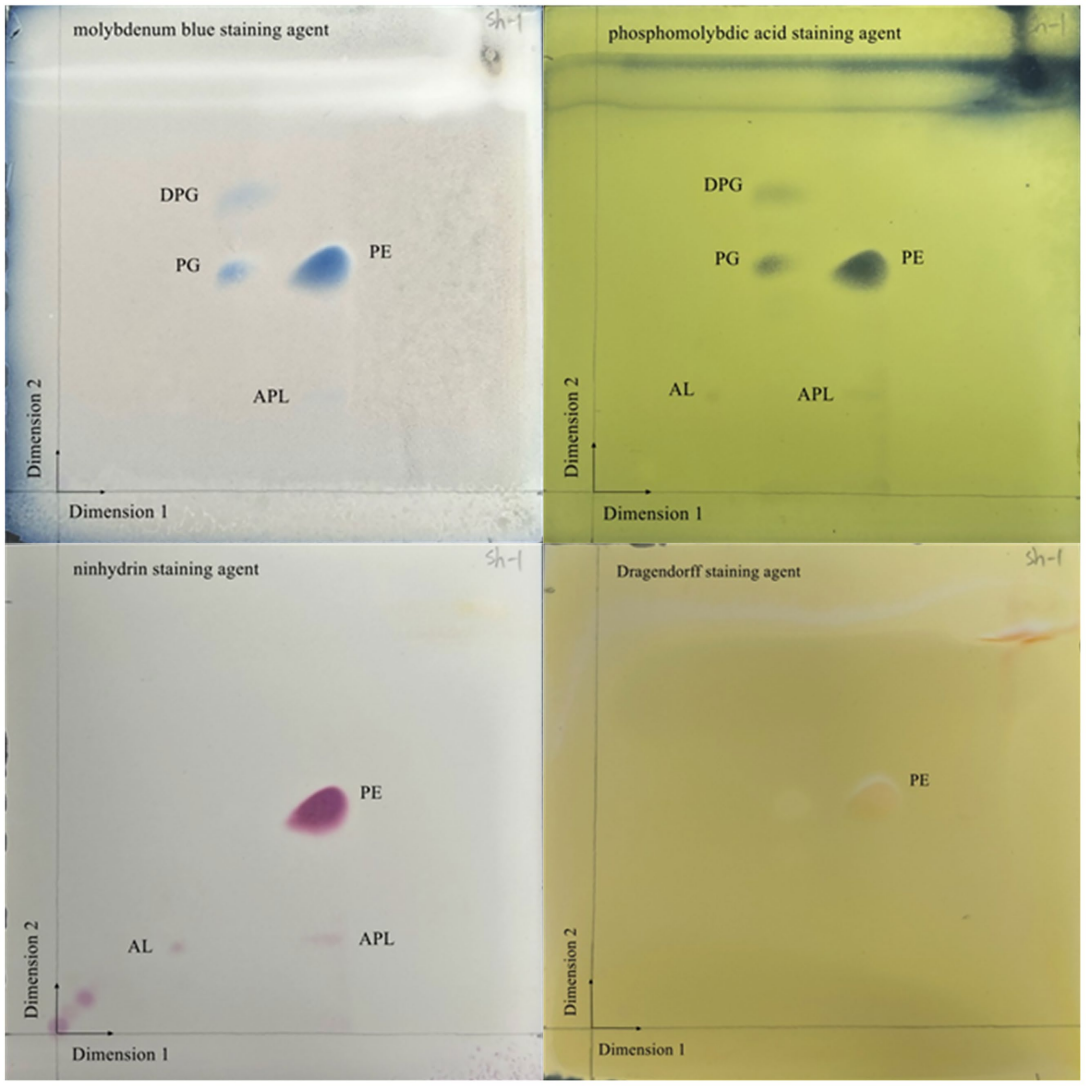
Two-dimensional TLC plate of polar lipids extracted from JZ001 strain. The plate was sprayed with 10% (v/v) molybdophosphoric aicd to show all polar lipids present. DPG, diphosphatidylglycerol; PG, phosphatidylglycerol; PE, phosphatidylethanolamine; AL, aminolipid; APL, aminophospholipid.

The cellular fatty acid and polar lipid profiles of strain JZ001 were determined (Table 2; Figure 3). Using a 10% threshold to define predominant fatty acids, JZ001 exhibited the following: 12:0 (11.08%), 14:0 (12.00%), 16:0 (21.55%), 17:0 cyclo (13.99%), 12:0 aldehydes (22.32%), and an unknown component (10.95%). Polar lipid profiling revealed the presence of diphosphatidylglycerol, phosphatidylglycerol, phosphatidylethanolamine, and aminolipids. However, aminophospholipids were not detected (Figure 3). No novel fatty acid species, previously unreported in human *Shigella* strains, were detected in the JZ001 strain. Its core fatty acid profile remains consistent with the genus-level characteristics of *Shigella* (e.g., dominated by 16:0 and 17:0 cyclo).

**Table 2:** Cellular fatty acids and polar acid compositions.

Peak Name	Percent	Comment 1	Comment 2
10:0	0.13		
12:0	11.08		
11:0 3OH	0.10		
13:0	0.26		
12:0 3OH	0.15		
14:0 anteiso	0.04		
14:0	12.00		
15:0 anteiso	0.05		
15:1 ω8c	0.05		
16:0 iso	0.05		
16:1 ω5c	0.18		
16:0	21.55		
15:0 2OH	0.04		
17:1 iso ω5c	0.04		
15:0 3OH	0.13		
17:0 iso	0.06		
17:1 ω7c	0.09		
17:0 cyclo	13.99		
17:0	0.27		
16:0 3OH	0.15		
18:0	0.22		
19:0 iso	0.40		
19:0 cyclo ω8c	4.43		
19:0	0.38		
Summed Feature 2	22.32	12:0 aldehyde	unknoωn 10.9525
Summed Feature 3	5.76	16:1 ω7c/16:1 ω6c	16:1 ω6c/16:1 ω7c
Summed Feature 5	0.15	18:0 ante/18:2 ω6,9c	18:2 ω6,9c/18:0 ante
Summed Feature 8	5.92	18:1 ω7c	18:1 ω6c

### General features of the JZ001 genome

Whole-genome sequencing of strain JZ001 produced 8,361,226 clean reads, totaling 1,249,861,482 base pairs. The average read length was 12,933.9 bp, with the longest read measuring 22,023 bp. The total assembled sequence length reached 1,761,547,250 bp, corresponding to 98.82% genome coverage, ensuring high sequencing accuracy. Raw data have been deposited in the NCBI database under BioProject number PRJNA1238207.

**Figure 4:**
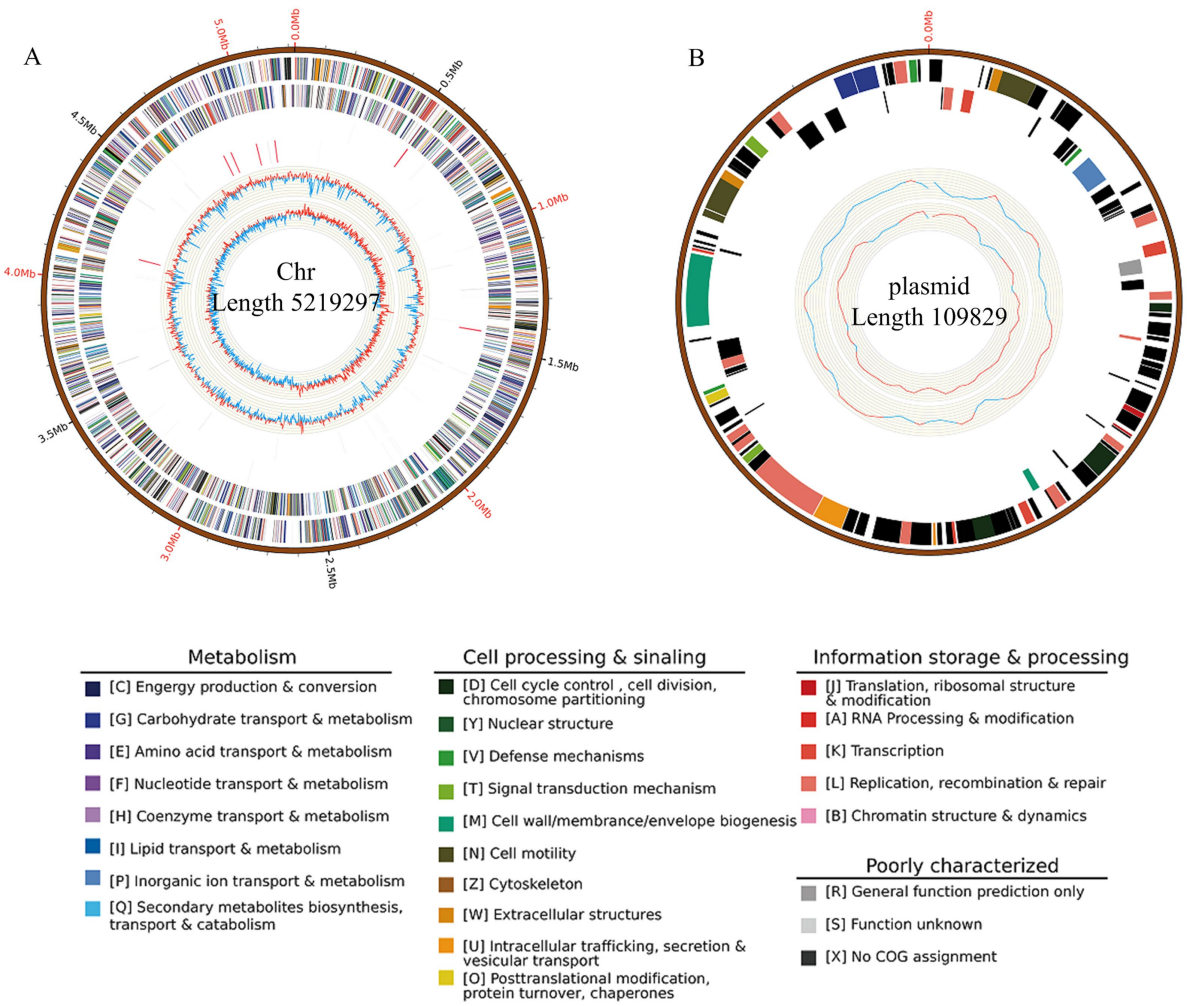
Circular map of the JZ001 genome. The genome is composed of a chromosome and one plasmid. The outermost ring represents the scale marks of the genome. The two following rings within the scale ring depict coding regions (CDSs) in the forward (blue) and reverse (yellow) strand. Moving toward the center, the next rings depict the rRNA (red) in the forward and reverse strand, followed by rings showing the tRNAs (black) in the forward and reverse strands. The innermost ring shows the GC content. The figure was generated by Circos software.

Genome assembly resulted in two gap-free, circular contigs. The larger contig, with a total length of 5,219,297 bp (Figure 4A; Table 3), corresponds to the chromosomal replicon. The smaller contig measured 109,829 bp and likely represents the virulence plasmid (Figure 4B).

**Table 3:** Basic genome information of JZ001.

Genomic contents	Chromosome	Plasmid
Number of ORFs	5,282	
Genome size (bp)	5,219,297	109,829
G + C (%)	50.65	40.14
Genomic islands (number of genes/%)	24	
Annotated proteins by Swiss-Prot database		
Number genes assigned to COG categories	2,274	
Number genes predicted as VFDB		
Number of rRNAs (16 s, 5 s, 23 s)	7,8,7	
Number of tRNAs	91	
Number of sRNAs	199	
CRISPR-associated genes	0	

ORF, open reading frames; COG, cluster of orthologous groups; VFDB, Database of Virulence Factors of Pathogenic Bacteria.

### Mobile genetic elements

**Figure 5:**
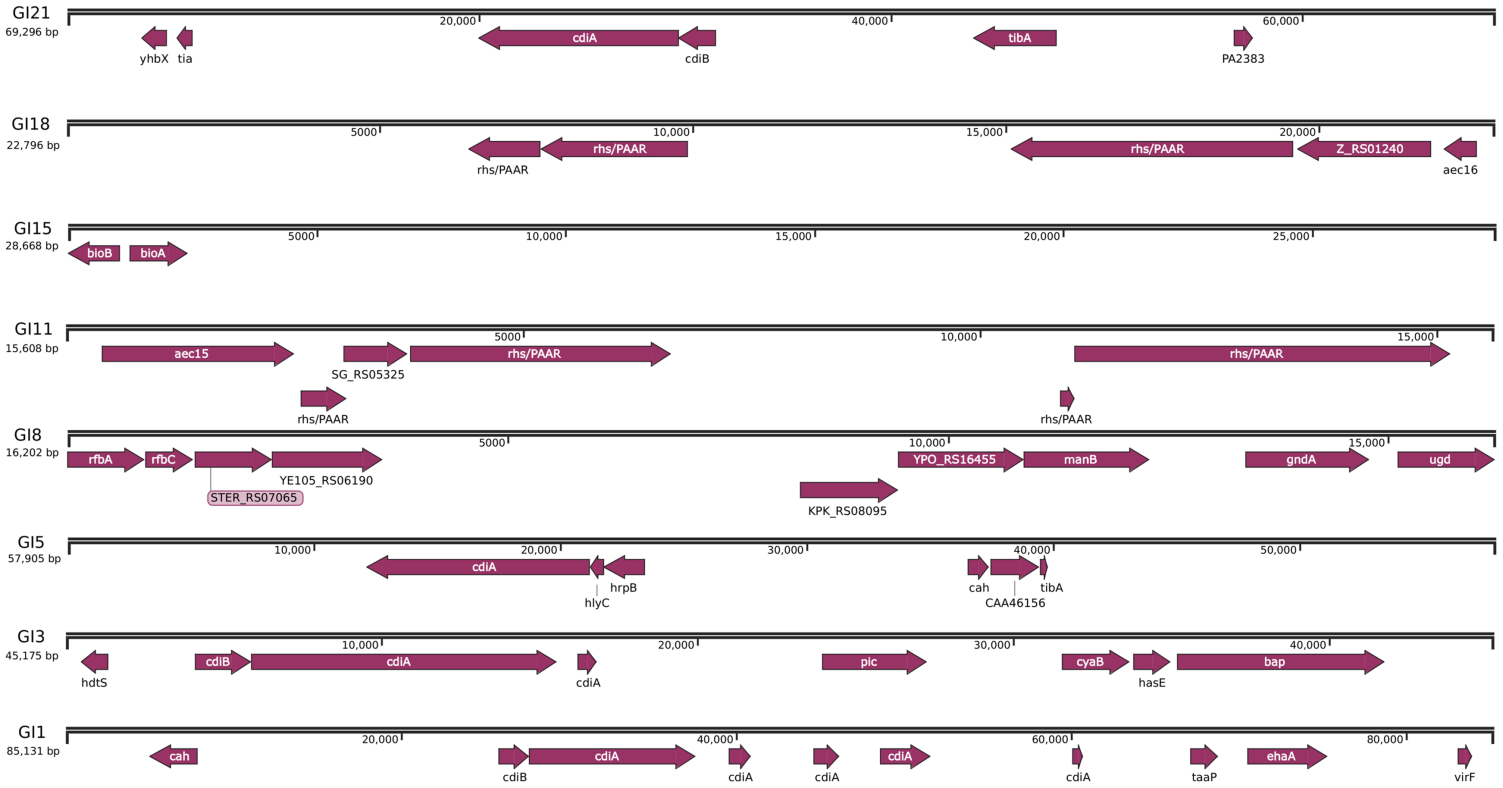
Detailed mapping pathogenicity islands and plasmid in JZ001 genome. Schematic representation of the genes carried within the eight SPIs predicated in the genome of JZ001. The grey boxes represented the range of SPIs, and the brown boxes showed the CDS encoded in these SPIs regions.

To further elucidate the genomic basis of adaptation and virulence, mobile genetic elements in JZ001 were analyzed. The chromosome of JZ001 was predicted to contain 24 genomic islands (GIs), designated GI1–GI24 (Table 4). Several of these GIs were associated with virulence and included SPI-1, SPI-3, SPI-5, SPI-8, SPI-11, SPI-15, SPI-18, and SPI-21. Their structures and functions are illustrated in Figure 5. GI 21 of this strain harbors the cidA and tib genes, whereas GI 18 contains four core genes: rfbA, rfbC, gndA, and ugd. GI 5 carries cidA, hrpB, tibA, and cah. GI 3 is enriched in multiple genes related to virulence and metabolism, including cidA, cyaB, and pic. Here, cyaB acts as a core effector gene contributing to pathogenicity, while picA and picB encode serine proteases. GI 1 predominantly carries the cidA and ehaG genes.

**Table 4:** Predicated pathogenicity islands (SPIs) in ZJ001.

SPIs	Position	GI Length	Gene name	Function
GI1	47,708…132,838	85,131	–	
GI2	862,393…884,609	22,217	–	
GI3	96,474…941,648	45,175	acpP	Acyl carrier protein; putative acyl carrier protein (A)
GI4	1,275,671…1,306,677	31,007	–	
GI5	1,314,694…1,372,598	57,905	ISSfl3 orfC (A)	Transposase
GI6	1,380,778…1,410,476	29,699	–	
GI7	1,860,774…1,863,529	2,756	–	
GI8	2,039,634…2,055,835	16,202	rfbA, rmlA, rffH	Glucose-1-phosphate thymidylyltransferase [EC:2.7.7.24]; glucose-1-phosphate thymidylyltransferase (A)
GI9	2,130,179…2,161,346	31,168	iraM, anti-adapter protein IraM	Conserved predicted protein (A)
GI10	2,351,132…2,359,335	8,204	hns	DNA-binding transcriptional dual regulator H-NS (A)
GI11	2,556,636…2,572,243	15,608	–	
GI12	2,721,346…2,723,846	2,501	dicC, dicB	Transcriptional repressor of cell division inhibition gene; Repressor protein of division inhibition gene
GI13	2,991,531…3,001,405	9,875	–	
GI14	3,019,353…3,032,431	13,079	–	
GI15	3,470,555…3,499,222	28,668	bioF, bioF	8-amino-7-oxononanoate; synthase8-amino-7-oxononanoate synthase (A)
GI16	3,587,880…3,597,144	9,265	–	
GI17	4,031,885…4,044,836	12,952	yagS, yagS	Xanthine dehydrogenase YagS FAD-binding subunit; predicted oxidoreductase (A)
GI18	4,070,926…4,093,721	22,796	NIT2, yafV	Omega-amidase; C-N hydrolase family amidase (A)
GI19	4,469,415…4,486,107	16,693	mcrB	5-methylcytosine-specific restriction enzyme B; putative ATPase family associated with various cellular activities
GI20	4,574,242…4,586,331	12,090	yjfL	Putative membrane protein; domain-containing inner membrane protein YjfL (A)
GI21	4,625,409…4,694,704	69,296	–	
GI22	5,172,261…5,207,233	34,973	–	
GI23	30,373…48,430	18,058	–	
GI24	76,887…87,540	10,654	–	

Prophages temperate phage genomes integrated into the bacterial chromosome are known to enhance bacterial adaptability and adhesion. PHAST analysis identified eight prophage regions in JZ001, totaling 368,421 bp, with an average length of 46,052.62 bp. Details of these prophages, including length, genomic position, number of CDSs, and GC content, are presented in Table 5.

**Table 5:** Prophage content of *Shigella* sp. (JZ001) analyzed using PHAST tool.

Region	Length (Kb)	GC content (%)	Begin_region	End_region	Accession number
1	63,658	48.78	1,345,904	1,409,561	NC_001416
2	42,132	50.24	2,114,309	2,156,440	NC_003356
3	27,489	48.89	2,520,478	2,547,966	NC_008562
4	50,473	49.39	2,680,010	2,730,482	NC_001416
5	47,226	48.48	2,991,531	3,038,756	NC_004827
6	36,714	51.53	3,139,532	3,102,819	NC_009382
7	65,756	47.65	3,473,892	3,539,647	NC_002730
8	34,973	54.22	5,172,261	5,207,233	NC_009237

### Comparative genome analysis

**Figure 6:**
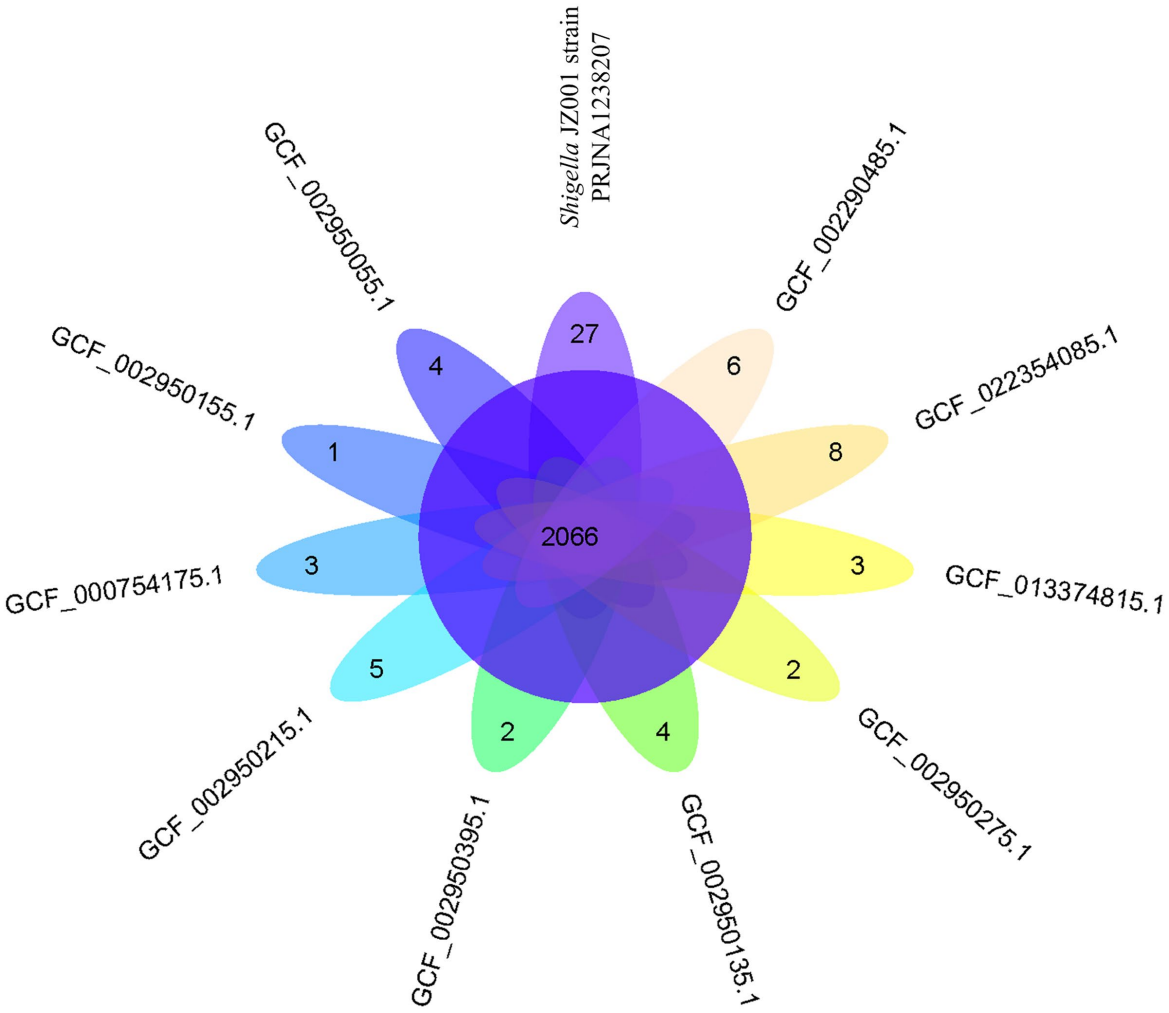
Venn diagram showing the number of genes of orthologous CDSs shared between ten strains and *Shigella* JZ001 strain (PRJNA1238207).

**Figure 7:**
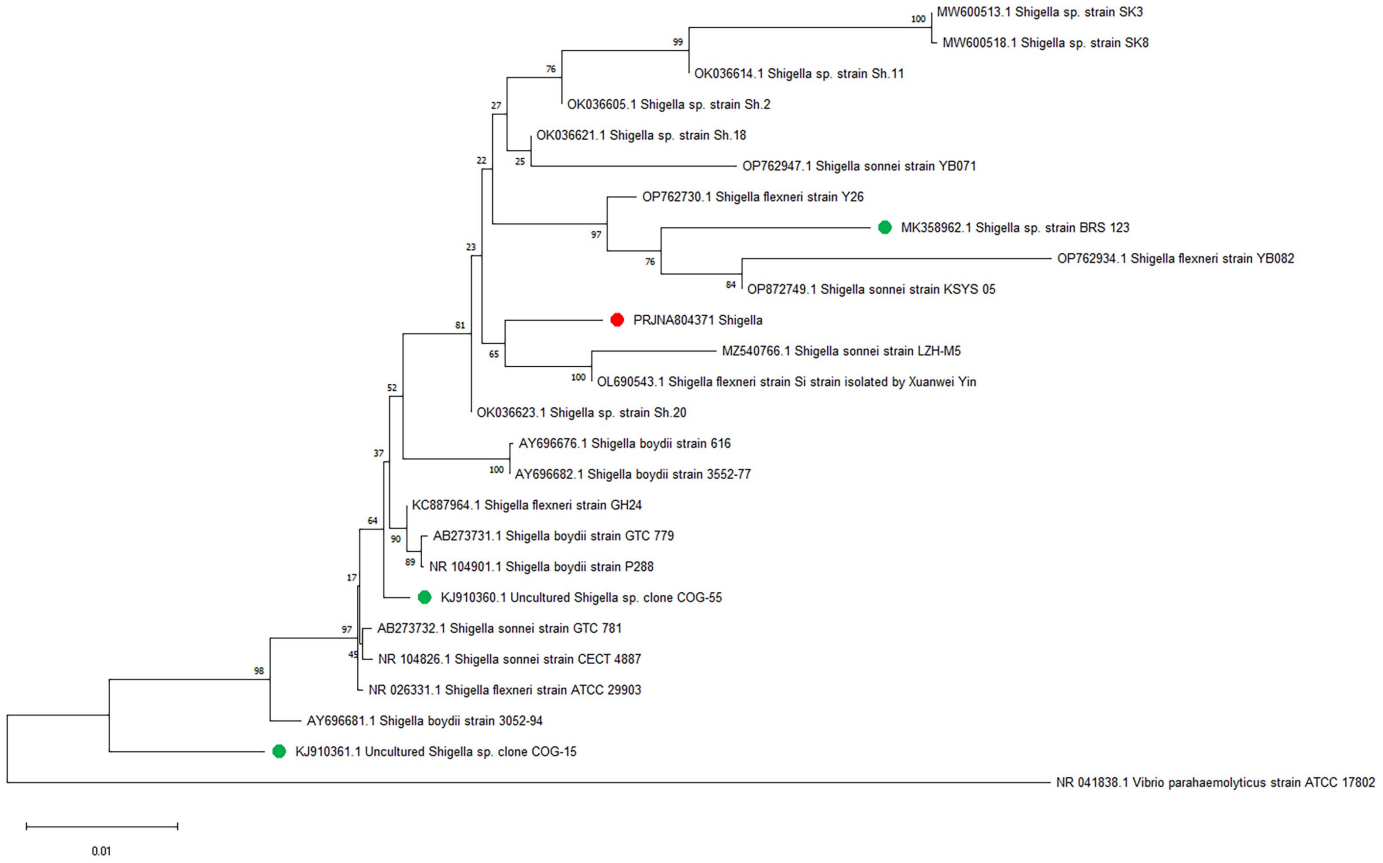
Phylogenetic tree depicting the relationship between JZ001 and 25 reference strains constructed based on the genomic BLAST in NCBI.

Whole-genome alignment was performed between strain JZ001 and ten publicly available *Shigella* spp. genomes. Given that no mouse-derived *Shigella* strain data is currently accessible in public data repositories; these ten genomes were selected from the ATCC database of human strains and chosen with 1–2 representative strains per *Shigella* serogroup to encompass taxonomic diversity. Homologous genomic blocks shared among these strains were identified, indicating a high degree of similarity (Figure 6; Table 6). ANI (Average Nucleotide Identity) analysis revealed that JZ001 had the highest similarity to *Shigella sonnei* strain SE6-1 (ANI > 99%). Phylogenetic analysis based on 16S rRNA sequences using the neighbor-joining method revealed that the strains clustered according to their serotypes. Notably, strain JZ001 exhibited close phylogenetic relatedness to *Shigella sonnei* (GenBank accession: MZ540766.1) (Figure 7).

**Table 6:** Basic features of ten strains and ANI value between these strains with JZ001.

Strain	Description	Size	GC%	ANI Value with JZ001
JZ001	Mice strain	5,3,291,26	50.65	100
GCF_002950055.1	*Shigella dysenteriae* strain ATCC	4,880,735	51	96.671
GCF_002950135.1	*Shigella boydii* strain ATCC	5,106,737	51	97.5445
GCF_002950155.1	*Shigella dysenteriae* strain ATCC	4,716,399	51	97.6489
GCF_002950215.1	*Shigella flexneri* 2a strain	4,659,463	51	97.5195
GCF_002950275.1	*Shigella boydii* strain ATCC	4,575,738	51	97.6135
GCF_002950395.1	*Shigella sonnei* strain ATCC	4,975,028	51	98.0446
GCF_013374815.1	*Shigella sonnei* strain SE6-1	4,762,774	50.5	99.0537
GCF_022354085.1	*Shigella dysenteriae* strain	5,075,418	50.5	97.6598

## Discussion

*Shigella* spp., are a group of Gram-negative enterobacteria characterized by a lack of flagellar motility and an inability to ferment lactose (2). S*higella* has no known animal reservoirs and is therefore considered to be human specific. Previous studies have primarily used mice to model infection human shigellosis infection, using intraperitoneal challenge with *Shigella* to mimic human bacillary dysentery (4).

Although mice are used to model *Shigella* infectious diseases, they are not susceptible to *Shigella* (34, 35). Mouse gut microbiota provides a protective barrier against human *Shigella* (36). Some *Shigella* effectors required for colonization specifically target human proteins and are often unable to interact with the mouse analogs (36). Therefore, the isolation of murine *Shigella* and the use of mouse *Shigella* to establish an infection model may provide more favorable model tools for subsequent research on the biological properties, pathogenicity, and other aspects of *Shigella* (4). However, we found no reports on the complete genome and biological characteristics of shigellosis in mice. In this research we isolate a murine *Shigella* sp. JZ001, for the first time, and sequenced the complete genome of and analysed the biological characteristics of the murine *Shigella* spp.

Human-derived *Shigella* strains primarily colonize the colonic mucosa, where glucose serves as the dominant carbon source in their ecological niche (37, 38). These isolates universally lack the ability to metabolize D-maltose and D-trehalose (39), and they typically cannot efficiently metabolize D-fructose or D-galactose. Moreover, most human-derived *Shigella* strains do not ferment D-mannose (40), with only a few serotypes showing weak metabolic activity toward this substrate.

In contrast, strain JZ001isolated from mice in the present study has retained and optimized multiple carbon metabolic pathways through natural selection. Beyond *α*-D-glucose, JZ001 can metabolize D-maltose, D-trehalose, D-mannose, D-fructose, D-galactose, and propionic acid. This suggests that JZ001 possesses a more versatile monosaccharide metabolic system and a broader range of metabolic substrates. Notably, it can utilize not only carbohydrates but also short-chain fatty acids (e.g., propionic acid) as alternative carbon and energy sources.

Additional key phenotypes of JZ001 include tolerance to acidic conditions and high osmotic stress, traits that enhance its ability to traverse the gastric acid barrier and increase its survival in feces. Importantly, the JZ001 strain exhibits a distinct antimicrobial resistance profile, characterized by resistance to rifampicin and vancomycin. Early literature reports have demonstrated that *Shigella flexneri* and *Shigella sonnei* isolates of human origin were 100% susceptible to rifampicin (41). However, in recent years, rifampicin-resistant strains of human-derived *Shigella* have also been documented, which are attributed to genetic mutations (42). In contrast, the intrinsic resistance of the JZ001 strain to vancomycin is consistent with the well-established vancomycin resistance phenotype of human-derived *Shigella* strains reported in previous studies (43).

As core components of the cell membrane, polar lipids and fatty acids can influence critical bacterial characteristics such as virulence, environmental adaptability, and drug susceptibility (37). The major lipidomic difference between strain JZ001 and previously reported human *Shigella* strains is the absence of phosphatidylserine (PS) in JZ001. This absence represents a distinguishing signature feature of the strain. Future experiments will compare the virulence of JZ001 with that of human *Shigella* strains using cell invasion assays and animal models, with the aim of elucidating the molecular mechanisms underlying this difference and its biological significance.

The pathogenesis of *Shigella* spp. is strictly dependent on the virulence plasmid, which encodes several factors that are essential for invasion and subversion of host defenses (44). *Shigella* genomes naturally harbor hundreds of insertion sequences (IS), and their genes are frequently inactivated either through IS-mediated disruption or IS-mediated genome rearrangement. In addition, the insertion sequences (IS) elements in *Shigella* have been shown to contribute to the antibiotic resistance and pathogen evolution (45, 46).

Strain JZ001 harbors functional genes within its genomic islands (GIs). As shown in Figure 5, genomic island GI21 contains cidA and tibA. Previous studies have reported that the cidA gene encodes a contact-dependent inhibition (CDI) protein (47), which suppresses the growth of other commensal bacteria in the intestine through intercellular contact, thereby providing *Shigella* with an ecological advantage in gut microbiota competition. The adhesion encoded by the tibA gene enhances bacterial adherence to intestinal epithelial cells, facilitating infection and colonization (48).

Genomic island GI18 contains three core genes rfbC, gndA, and ugd, that contribute to bacterial metabolic survival and structural resistance. Specifically, rfbC is involved in lipopolysaccharide (LPS) biosynthesis, a key component of the *Shigella* cell wall that increases resistance to host immune pressures in the intestine (49). gndA encodes glucose-6-phosphate dehydrogenase, which generates NADPH for bacterial survival via the pentose phosphate pathway (PPP) (38). However, the virulence potential of strain JZ001 requires further experimental validation, including comparative studies of virulence-associated genes between murine- and human-derived *Shigella* strains. Based on the findings of this study, we hypothesize that the core genomic islands of strain JZ001 carry functional genes related to adhesion, barrier penetration, virulence regulation, and metabolic adaptation, which may represent key genetic elements enabling pathogenicity in suckling mouse hosts.

Comparative genomic analysis revealed that JZ001 shares >99% average nucleotide identity (ANI) with *Shigella sonnei* strain SE6-1 (MZ540766.1), strongly supporting its tentative classification as *S. sonnei.* ANI is widely used to assess evolutionary relationships and species boundaries, with values >95% indicating that two genomes belong to the same species (50, 51). JZ001 and *S. sonnei* clearly exceed this threshold, providing robust genomic evidence to classify JZ001 as a member of *S. sonnei*. A notable limitation, however, is the difference in host origin: JZ001 was isolated from mice, whereas all reference genomes (including SE6-1) are human-derived. Because *Shigella* is traditionally regarded as a human-specific pathogen, and there are currently no literature reports on naturally occurring *Shigella* isolates from mice, it remains unclear whether JZ001 represents a mouse-adapted variant of *S. sonnei*. Future studies should compare the transcriptomic profiles of JZ001 during murine infection with those of human-derived *S. sonnei* strains to identify host-specific gene expression signatures.

In conclusion, the genome sequence reported here represents the first complete genome sequence of a mouse-derived *Shigella* strain. It provides a high-quality reference genome that will be extremely valuable for transcriptomics, differential expression analysis, studies of molecular pathogenesis, and investigations into genome evolution. Subsequent studies will establish a suckling mouse infection model for strain JZ001 to enable dynamic monitoring of microbial alterations, host inflammatory responses, and disease outcomes. In addition, gene knockout and transcriptomic analyses will be performed to determine whether JZ001 exhibits pathogenicity in mice.

## Data Availability

The datasets presented in this study can be found in online repositories. The names of the repository/repositories and accession number(s) can be found at: https://www.ncbi.nlm.nih.gov/, PRJNA1238207.

## Footnotes

^1^ https://www.megasoftware.net/

## References

[1] Bengtsson RJ Simpkin AJ Pulford CV Low R Rasko DA Rigden DJ . Pathogenomic analyses of *Shigella* isolates inform factors limiting shigellosis prevention and control across LMICs. Nat Microbiol. (2022) 7:251–61. doi: 10.1038/s41564-021-01054-z, PMID: 35102306 PMC8813619

[2] Baker S The HC. Recent insights into *Shigella*. Curr Opin Infect Dis. (2018) 31:449–54. doi: 10.1097/qco.0000000000000475, PMID: 30048255 PMC6143181

[3] GBD Diarrhoeal Diseases Collaborators. Estimates of global, regional, and National Morbidity, mortality, and aetiologies of diarrhoeal diseases: a systematic analysis for the global burden of disease study 2015. Lancet Infect Dis. (2017) 17:909–48. doi: 10.1016/s1473-3099(17)30276-1 28579426 PMC5589208

[4] Alphonse N Odendall C. Animal models of shigellosis: a historical overview. Curr Opin Immunol. (2023) 85:102399. doi: 10.1016/j.coi.2023.102399, PMID: 37952487

[5] Maurelli AT Routh PR Dillman RC Ficken MD Weinstock DM Almond GW . *Shigella* infection as observed in the experimentally inoculated domestic pig, *Sus scrofa domestica*. Microb Pathog. (1998) 25:189–96. doi: 10.1006/mpat.1998.0230, PMID: 9817822

[6] Onyango DM Wandili S Kakai R Waindi EN. Isolation of Salmonella and *Shigella* from fish harvested from the Winam gulf of Lake Victoria, Kenya. J Infect Dev Ctries. (2009) 3:99–104. doi: 10.3855/jidc.56 19755738

[7] Shi R Yang X Chen L Chang HT Liu HY Zhao J . Pathogenicity of *Shigella* in chickens. PLoS One. (2014) 9:e100264. doi: 10.1371/journal.pone.0100264, PMID: 24949637 PMC4064985

[8] Zhu Z Shi Y Zhou X Li B Zhang J. Molecular characterization of fluoroquinolone and/or cephalosporin resistance in *Shigella Sonnei* isolates from yaks. BMC Vet Res. (2018) 14:177. doi: 10.1186/s12917-018-1500-6, PMID: 29879965 PMC5992640

[9] Zhu Z Wang W Cao M Zhu Q Ma T Zhang Y . Virulence factors and molecular characteristics of *Shigella flexneri* isolated from calves with Diarrhea. BMC Microbiol. (2021) 21:214. doi: 10.1186/s12866-021-02277-0, PMID: 34271864 PMC8285881

[10] Brunner K Samassa F Sansonetti PJ Phalipon A. *Shigella*-mediated immunosuppression in the human gut: subversion extends from innate to adaptive immune responses. Hum Vaccin Immunother. (2019) 15:1317–25. Epub 2019419. doi: 10.1080/21645515.2019.1594132, PMID: 30964713 PMC6663138

[11] Zhao W Yu ML Tao X Cheng MH Liu CC Liu Y . Analysis of the intestinal microbial community altered during rotavirus infection in suckling mice. Virol J. (2021) 18:254. doi: 10.1186/s12985-021-01727-5, PMID: 34930341 PMC8686622

[12] Wang W Cao J Li JR Yang F Li Z Li LX. Comparative analysis of the gastrointestinal microbial communities of Bar-headed goose (*Anser indicus*) in different breeding patterns by high-throughput sequencing. Microbiol Res. (2016) 182:59–67. doi: 10.1016/j.micres.2015.10.003, PMID: 26686614

[13] Yasuda K Oh K Ren B Tickle TL Franzosa EA Wachtman LM . Biogeography of the intestinal mucosal and Lumenal microbiome in the Rhesus macaque. Cell Host Microbe. (2015) 17:385–91. doi: 10.1016/j.chom.2015.01.015, PMID: 25732063 PMC4369771

[14] Panchalingam S Antonio M Hossain A Mandomando I Ochieng B Oundo J . Diagnostic microbiologic methods in the GEMS-1 case/control study. Clin Infect Dis. (2012) 55 Suppl 4:S294–302. doi: 10.1093/cid/cis754, PMID: 23169941 PMC3502308

[15] Kumar S Stecher G Li M Knyaz C Tamura K. MEGA X: molecular evolutionary genetics analysis across computing platforms. Mol Biol Evol. (2018) 35:1547–9. doi: 10.1093/molbev/msy096, PMID: 29722887 PMC5967553

[16] Chin CS Alexander DH Marks P Klammer AA Drake J Heiner C . Nonhybrid, finished microbial genome assemblies from long-read SMRT sequencing data. Nat Methods. (2013) 10:563–9. doi: 10.1038/nmeth.2474, PMID: 23644548

[17] Berlin K Koren S Chin CS Drake JP Landolin JM Phillippy AM. Assembling large genomes with single-molecule sequencing and locality-sensitive hashing. Nat Biotechnol. (2015) 33:623–30. doi: 10.1038/nbt.3238, PMID: 26006009

[18] Lowe TM Eddy SR. tRNAscan-SE: a program for improved detection of transfer RNA genes in genomic sequence. Nucleic Acids Res. (1997) 25:955–64. doi: 10.1093/nar/25.5.955, PMID: 9023104 PMC146525

[19] Lagesen K Hallin P Rødland EA Staerfeldt HH Rognes T Ussery DW. RNAmmer: consistent and rapid annotation of ribosomal RNA genes. Nucleic Acids Res. (2007) 35:3100–8. doi: 10.1093/nar/gkm160, PMID: 17452365 PMC1888812

[20] Petersen TN Brunak S von Heijne G Nielsen H. SignalP 4.0: discriminating signal peptides from transmembrane regions. Nat Methods. (2011) 8:785–6. doi: 10.1038/nmeth.1701, PMID: 21959131

[21] Arndt D Marcu A Liang Y Wishart DS. PHAST, PHASTER and PHASTEST: tools for finding prophage in bacterial genomes. Brief Bioinform. (2019) 20:1560–7. doi: 10.1093/bib/bbx121, PMID: 29028989 PMC6781593

[22] Bertelli C Brinkman FSL. Improved Genomic Island predictions with Islandpath-Dimob. Bioinformatics. (2018) 34:2161–7. doi: 10.1093/bioinformatics/bty095, PMID: 29905770 PMC6022643

[23] Ge R Mai G Wang P Zhou M Luo Y Cai Y . CRISPRdigger: detecting CRISPRs with better direct repeat annotations. Sci Rep. (2016) 6:32942. doi: 10.1038/srep32942, PMID: 27596864 PMC5011713

[24] Almagro Armenteros JJ Tsirigos KD Sønderby CK Petersen TN Winther O Brunak S . SignalP 5.0 improves signal peptide predictions using deep neural networks. Nat Biotechnol. (2019) 37:420–3. doi: 10.1038/s41587-019-0036-z 30778233

[25] Juncker AS Willenbrock H Von Heijne G Brunak S Nielsen H Krogh A. Prediction of lipoprotein signal peptides in gram-negative Bacteria. Protein Sci. (2003) 12:1652–62. doi: 10.1110/ps.0303703, PMID: 12876315 PMC2323952

[26] Krogh A Larsson B von Heijne G Sonnhammer EL. Predicting transmembrane protein topology with a hidden Markov model: application to complete genomes. J Mol Biol. (2001) 305:567–80. doi: 10.1006/jmbi.2000.4315, PMID: 11152613

[27] Yu NY Wagner JR Laird MR Melli G Rey S Lo R . PSORTb 3.0: improved protein subcellular localization prediction with refined localization subcategories and predictive capabilities for all prokaryotes. Bioinformatics. (2010) 26:1608–15. doi: 10.1093/bioinformatics/btq249, PMID: 20472543 PMC2887053

[28] Urban M Cuzick A Seager J Wood V Rutherford K Venkatesh SY . PHI-base: the pathogen-host interactions database. Nucleic Acids Res. (2020) 48:D613–20. doi: 10.1093/nar/gkz904, PMID: 31733065 PMC7145647

[29] Chen L Yang J Yu J Yao Z Sun L Shen Y . VFDB: a reference database for bacterial virulence factors. Nucleic Acids Res. (2005) 33:D325–8. doi: 10.1093/nar/gki008, PMID: 15608208 PMC539962

[30] Krzywinski M Schein J Birol I Connors J Gascoyne R Horsman D . Circos: an information aesthetic for comparative genomics. Genome Res. (2009) 19:1639–45. doi: 10.1101/gr.092759.109, PMID: 19541911 PMC2752132

[31] Lee I Ouk Kim Y Park SC Chun J. OrthoANI: an improved algorithm and software for calculating average nucleotide identity. Int J Syst Evol Microbiol. (2016) 66:1100–3. doi: 10.1099/ijsem.0.000760, PMID: 26585518

[32] Marçais G Delcher AL Phillippy AM Coston R Salzberg SL Zimin A. MUMmer4: a fast and versatile genome alignment system. PLoS Comput Biol. (2018) 14:e1005944. doi: 10.1371/journal.pcbi.1005944, PMID: 29373581 PMC5802927

[33] Emms DM Kelly S. OrthoFinder: phylogenetic orthology inference for comparative genomics. Genome Biol. (2019) 20:238. doi: 10.1186/s13059-019-1832-y, PMID: 31727128 PMC6857279

[34] Freter R. Experimental enteric *Shigella* and Vibrio infections in mice and guinea pigs. J Exp Med. (1956) 104:411–8. doi: 10.1084/jem.104.3.411, PMID: 13357693 PMC2136576

[35] McGuire CD Floyd TM. Studies on experimental shigellosis. I. *Shigella* infections of normal mice. J Exp Med. (1958) 108:269–76. doi: 10.1084/jem.108.2.269 13563761 PMC2136868

[36] Mitchell PS Roncaioli JL Turcotte EA Goers L Chavez RA Lee AY . NAIP-NLRC4-deficient mice are susceptible to shigellosis. eLife. (2020) 9:9. doi: 10.7554/eLife.59022, PMID: 33074100 PMC7595732

[37] Tinevez JY Arena ET Anderson M Nigro G Injarabian L André A . *Shigella*-mediated oxygen depletion is essential for intestinal mucosa colonization. Nat Microbiol. (2019) 4:2001–9. doi: 10.1038/s41564-019-0525-3, PMID: 31383999 PMC6817363

[38] Waligora EA Fisher CR Hanovice NJ Rodou A Wyckoff EE Payne SM. Role of intracellular carbon metabolism pathways in *Shigella flexneri* virulence. Infect Immun. (2014) 82:2746–55. doi: 10.1128/iai.01575-13, PMID: 24733092 PMC4097621

[39] Ito H Kido N Arakawa Y Ohta M Sugiyama T Kato N. Possible mechanisms underlying the slow lactose fermentation phenotype in *Shigella* Spp. Appl Environ Microbiol. (1991) 57:2912–7. doi: 10.1128/aem.57.10.2912-2917.1991, PMID: 1746953 PMC183896

[40] Gross RJ Thomas LV Cheasty T Rowe B Lindberg AA. Four new provisional serovars of *Shigella*. J Clin Microbiol. (1989) 27:829–31. doi: 10.1128/jcm.27.5.829-831.1989, PMID: 2501349 PMC267438

[41] Galushko NA D'Iachenko AG Chemich ND D'Iachenko PA. Antibiotic resistance of Shigellae and rationale for Etiotropic therapy of Shigella infections. Zh Mikrobiol Epidemiol Immunobiol. (2005) 2:71–5. 15881945

[42] Ye C Lan R Xia S Zhang J Sun Q Zhang S . Emergence of a new multidrug-resistant serotype X variant in an epidemic clone of *Shigella flexneri*. J Clin Microbiol. (2010) 48:419–26. doi: 10.1128/JCM.00614-09 19955273 PMC2815595

[43] Fernandes MM Ivanova K Hoyo J Pérez-Rafael S Francesko A Tzanov T. Nanotransformation of vancomycin overcomes the intrinsic resistance of gram-negative Bacteria. ACS Appl Mater Interfaces. (2017) 9:15022–30. doi: 10.1021/acsami.7b00217, PMID: 28393523

[44] Buchrieser C Glaser P Rusniok C Nedjari H D'Hauteville H Kunst F . The virulence plasmid Pwr100 and the repertoire of proteins secreted by the type iii secretion apparatus of *Shigella flexneri*. Mol Microbiol. (2000) 38:760–71. doi: 10.1046/j.1365-2958.2000.02179.x, PMID: 11115111

[45] Wei J Goldberg MB Burland V Venkatesan MM Deng W Fournier G . Complete genome sequence and comparative genomics of *Shigella flexneri* serotype 2a strain 2457T. Infect Immun. (2003) 71:2775–86. doi: 10.1128/iai.71.5.2775-2786.2003, PMID: 12704152 PMC153260

[46] Prosseda G Di Martino ML Campilongo R Fioravanti R Micheli G Casalino M . Shedding of genes that interfere with the pathogenic lifestyle: the *Shigella* model. Res Microbiol. (2012) 163:399–406. doi: 10.1016/j.resmic.2012.07.004, PMID: 22824069

[47] Halvorsen TM Schroeder KA Jones AM Hammarlöf D Low DA Koskiniemi S . Contact-dependent growth inhibition (CDI) systems deploy a large family of polymorphic Ionophoric toxins for inter-bacterial competition. PLoS Genet. (2024) 20:e1011494. doi: 10.1371/journal.pgen.1011494, PMID: 39591464 PMC11630599

[48] Mitra S Saha DR Pal A Niyogi SK Mitra U Koley H. Hemagglutinating activity is directly correlated with colonization ability of *Shigella*e in suckling mouse model. Can J Microbiol. (2012) 58:1159–66. doi: 10.1139/w2012-095, PMID: 22978650

[49] Rajakumar K Jost BH Sasakawa C Okada N Yoshikawa M Adler B. Nucleotide sequence of the rhamnose biosynthetic operon of *Shigella Flexneri* 2a and role of lipopolysaccharide in virulence. J Bacteriol. (1994) 176:2362–73. doi: 10.1128/jb.176.8.2362-2373.1994, PMID: 8157605 PMC205360

[50] Hernández-Salmerón JE Moreno-Hagelsieb G. Fastani, mash and dashing equally differentiate between Klebsiella species. PeerJ. (2022) 10:e13784. doi: 10.7717/peerj.13784, PMID: 35891643 PMC9308963

[51] Jain C Rodriguez RL Phillippy AM Konstantinidis KT Aluru S. High throughput ANI analysis of 90k prokaryotic genomes reveals clear species boundaries. Nat Commun. (2018) 9:5114. doi: 10.1038/s41467-018-07641-9 30504855 PMC6269478

