# Supplementary material for: Whole genome analysis of Shigella sp. JZ001: a novel strain isolated from diarrheic suckling mice
Source: Front Vet Sci. 2025 Nov 21;12:1686554. doi: 10.3389/fvets.2025.1686554 (PMC12679885; doi:10.3389/fvets.2025.1686554)
Supplement: Supplementary file 1 [file Table_1.docx]

| **genus_tax** | RV-1 | RV-2 | RV-3 | RV-4 | RV-5 | RV-6 | NC-1 | NC-2 | NC-3 | NC-4 | NC-5 | NC-6 |
| --- | --- | --- | --- | --- | --- | --- | --- | --- | --- | --- | --- | --- |
| **g__Lactobacillus** | 0.423164 | 0.353823 | 0.154576 | 0.395314 | 0.260125 | 0.515725 | 0.630789 | 0.384789 | 0.39963 | 0.670596 | 0.676133 | 0.455351 |
| **g__Escherichia/Shigella** | 0.427248 | 0.596518 | 0.619716 | 0.389757 | 0.635652 | 0.335298 | 8.42E-05 | 0.136894 | 0.111633 | 0.02825 | 0.024693 | 0.000674 |
| **g__Fusobacterium** | 0.017514 | 0.005557 | 0.059932 | 0.011767 | 0.008273 | 0.05528 | 0.10496 | 0.094708 | 0.168407 | 0.085172 | 0 | 0.111906 |
| **g__Streptococcus** | 0.0032 | 0.002989 | 0.019662 | 0.007473 | 0.003642 | 0.019493 | 0.050459 | 0.052543 | 0.018609 | 0.024272 | 0.033029 | 0.04246 |
| **g__Helicobacter** | 0 | 0 | 0 | 0 | 0 | 0 | 0 | 0 | 0.010589 | 0.001874 | 0.001979 | 0.044691 |
| **g__Gemella** | 0.003937 | 0.0016 | 0.009389 | 0.005326 | 0.003305 | 0.005789 | 0.007705 | 0.010189 | 0.015893 | 0.003158 | 0.007999 | 0.007936 |
| **g__Bacteroides** | 0 | 0 | 2.11E-05 | 0 | 0 | 0 | 0.000147 | 0 | 0.000653 | 0.004084 | 0 | 0.004021 |
| **g__Enterococcus** | 0.001326 | 0 | 2.11E-05 | 0.002147 | 0.00221 | 0.002105 | 0 | 0 | 0 | 0 | 0 | 0.000168 |
| **g__Rhodococcus** | 0.000463 | 0.000653 | 0.005621 | 0.000253 | 0.000421 | 0.001916 | 0.000126 | 0 | 0.000947 | 0.000337 | 2.11E-05 | 0.005179 |
| **g__Alloprevotella** | 0 | 0 | 0 | 0 | 0 | 0 | 6.32E-05 | 0 | 0 | 0.000505 | 0 | 0.000105 |

Table 1S Relative percentage values of Microbial community composition of fecal samples from diarrheal suckling mice
